# Bi-directional associations between religious attendance and mental health: findings from a British birth cohort study

**DOI:** 10.1136/jech-2021-216943

**Published:** 2021-08-05

**Authors:** Aradhna Kaushal, Mai Stafford, Dorina Cadar, Marcus Richards

**Affiliations:** 1 Research Department of Behavioural Science and Health, University College London, London, UK; 2 The Health Foundation, London, UK; 3 MRC Unit for Lifelong Health and Ageing at UCL, University College London, London, UK

**Keywords:** mental health, epidemiology of ageing, longitudinal studies, cohort studies, aging

## Abstract

**Background:**

There is evidence that religious attendance is associated with positive outcomes for mental health; however, there are few longitudinal studies, and even fewer, which take into account the possibility of bi-directional associations. This study aimed to investigate bi-directional associations between religious attendance and mental health.

**Methods:**

Participants were 2125 study members who provided data at age 68–69 from the Medical Research Council National Survey of Health and Development (1946 British birth cohort study). Mental health was assessed using the 28-item General Health Questionnaire at ages 53, 60–64 and 68–69. Religious attendance was measured using a 4-point scale (weekly=3, monthly=2, less than monthly=1 or never=0) at ages 43, 60–64 and 68–69. Cross-lagged path analysis was used to assess reciprocal associations between mental health and religious attendance, adjusting for gender and education.

**Results:**

Previous religious attendance was strongly related to later attendance (r=0.62–0.74). Similarly, mental health at baseline was strongly associated with subsequent mental health scores (r=0.46–0.54). Poor mental health at age 53 and 60–64 was associated with more frequent religious attendance at age 60–64 (b=0.04; 95% CI: 0.02 to 0.06; p<0.05), and 68–69 (b=0.03; 95% CI: 0.02 to 0.06; p<0.05), respectively. There was no evidence that religious attendance at age 43, 60–64 or 68–69 was associated with later or concurrent mental health.

**Conclusion:**

Using birth cohort data from the UK, it was found that poor mental health was associated with later religious attendance but not vice versa. Future research should confirm these novel findings and explore the underlying mechanisms between religious attendance and mental health.

## Background

Depression and anxiety affect one in five people in the UK and are an increasingly important public health priority.^1^ Experiencing mental health difficulties can be accompanied by personal suffering, stigma and difficulties engaging with society, as well as a lower likelihood of employment, greater likelihood of poor physical health and lower life expectancy.^2^ In England, mental health is estimated to cost around £22.5 billion a year in health services and £28 billion in lost earnings and these costs are expected to increase by 45% by 2026.^3^

Several systematic reviews have found that religiosity, a term used to describe religious affiliation, beliefs, attendance at services and practices, has been associated with better mental health outcomes.^4 5^ A recent meta-analysis of 48 longitudinal studies built on these reviews by estimating the effect size of religiosity on mental health rather than the proportion of studies reporting a positive effect. The authors confirmed a positive effect of religiosity and spirituality on mental health, but found the total effect size was small (r=0.08).^6^ A limitation of these reviews is that due to the heterogeneity religiosity measures, it is difficult to know which aspects of religiosity are associated with mental health or account for the possibility that religious attendance and religious beliefs may impact health in different ways. A systematic review by Braam and Koenig focusing on 152 prospective studies found that just over half of the studies showed an inverse association between religiosity and depression, with the authors highlighting the need for research assessing the possibility of bi-directional associations.^7^ This review identified 12 studies that evaluated bi-directional associations, of which four studies suggest that depression is associated with a decrease in religion and spirituality with the remaining eight studies showing no association. Although most of the research on the associations between religiosity and mental health has been conducted in the USA, a recent study from the UK using household panel data suggest that religious attendance and beliefs are associated with subsequently better mental health and well-being.^8^

To date, there have been no investigations of the potential bi-directional relationship between religious involvement and mental health in the UK. The aim of this study was to investigate bi-directional associations between religious attendance and mental health using data from the Medical Research Council (MRC) National Survey of Health and Development (NSHD), also known as the British 1946 birth cohort. Based on previous research, we predicted that religious attendance would be associated with better mental health and that poor mental health would be related to a decrease in religious attendance.

## Methods

### Design and participants

**Figure 1:**
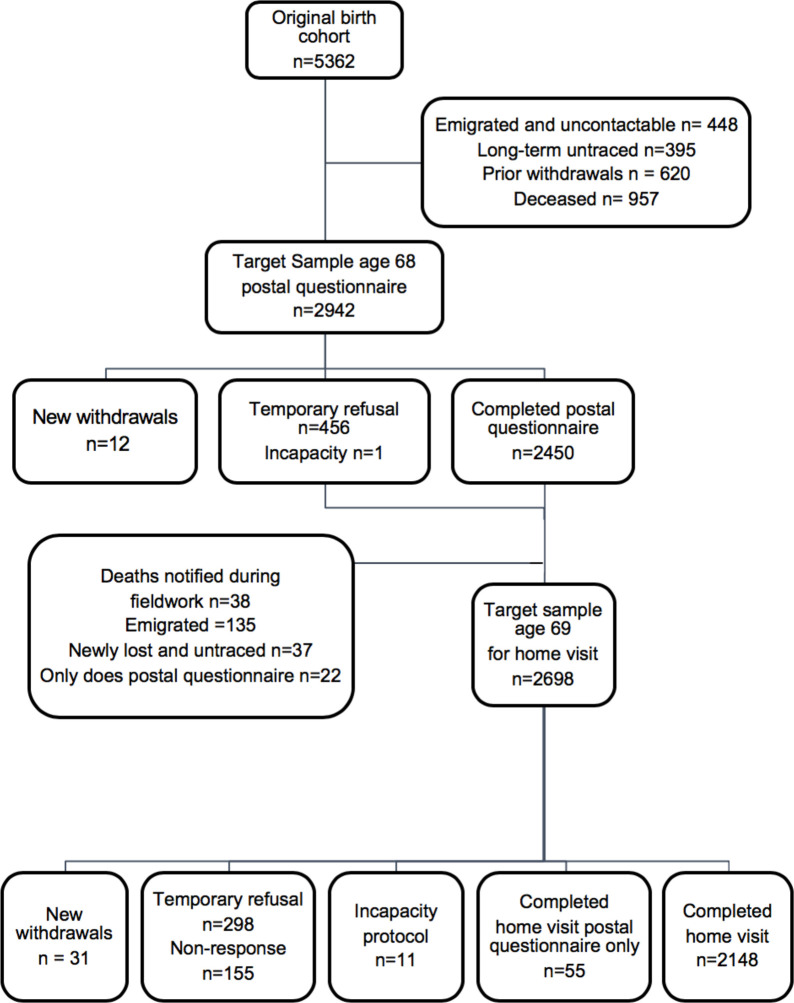
Target samples and response to the postal questionnaire and the home visit at age 68–69 in National Survey of Health and Development. Figure adapted from Kuh *et al.* 9

The MRC NSHD is a nationally representative British birth cohort selected from all births in 1 week of March 1946 and stratified by father’s social class. Originally consisting of 5362 single births to married women in England, Wales and Scotland, it has been followed up 24 times to date. Details of the data collection and response rates have been previously described.^9–11^ We included study members who took part in the most recent data collection at age 68–69 (n=2148). Details of the study members excluded from the analysis are shown in figure 1.

### Mental health

Symptoms of anxiety and depression were measured by a self-completed questionnaire at ages 53, 60–64 and 68–69 using the 28-item General Health Questionnaire (GHQ-28). The GHQ-28 contains four subscales; somatic symptoms, anxiety and insomnia, social dysfunction and severe depression.^12^ Each item was scored from 0 to 3, giving a minimum score of 0 and a maximum score of 84, with a higher score indicating higher psychological distress. The GHQ-28 had a skewed distribution in this sample and was log-transformed for use in the cross-lagged model.

### Religious attendance

Frequency of religious attendance was assessed at ages 43, 60–64 and 68–69 by postal questionnaire. The phrasing of this question and response options varied slightly between waves; at 43, they were asked if they helped to run church activities or participate in religious services, and at 60–64 and 68–69 they were asked how often they participated in church-related groups or religious activities. Response options were harmonised across waves, so that religious participation was categorised as ‘weekly’, ‘monthly’, ‘less often’ and ‘never’.

### Covariables

Models were adjusted for gender, and educational attainment as preliminary analysis showed these were confounding variables. Measures of social class were not included as these were not found to be associated with religious attendance above and beyond education (online supplemental table S1). Educational attainment was measured as the highest level of qualification obtained by age 26 based on the Burnham scale.^13^ This was grouped into no qualification, up to ordinary ('O') level (including vocational courses, sub General Certificate of Education (GCE)), advanced ('A') level or equivalent and higher (degree or higher).

10.1136/jech-2021-216943.supp1 Supplementary data

### Statistical analysis

**Figure 2:**
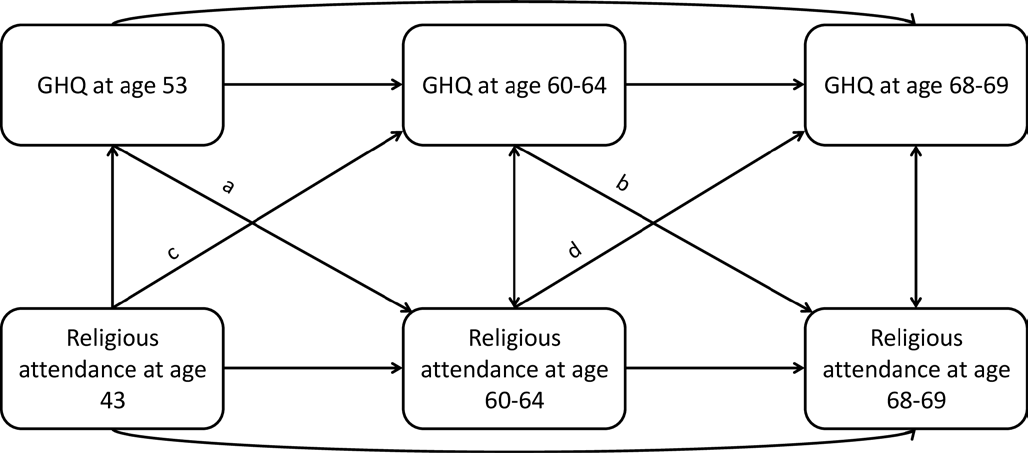
Conceptual auto-regressive cross-lagged model based on the repeat measures of religious attendance and mental health (GHQ) at ages 43/53, 60–64 and 68–69 in National Survey of Health and Development. GHQ, General Health Questionnaire.

An auto-regressive cross-lagged model was used to simultaneously assess reciprocal longitudinal associations between mental health and religious attendance over time using three-repeated measures for each of these variables (figure 2). This model can be used to test the direction of associations between two variables of interest with repeated measures. In this analysis, religious attendance reported at ages 43, 60–64 and 68–69 were analysed with mental health at ages 53, 60–64 and 68–69. The model was applied with equality constraints across waves (a=b and c=d) which is recommended if there is no loss of model fit (figure 2).^14^ Religious attendance (weekly=3, monthly=2, less than monthly=1 or never=0) and GHQ-28 score (0–84) were modelled as continuous variables. A sensitivity analysis modelling religious attendance as a categorical variable was conducted. Auto-correlations between religious attendance variables and mental health were calculated between all included waves of data collection.

The analysis was limited to participants who had complete data on GHQ-28 at age 68–69 (n=2125). Missing data on all other variables were addressed using full information maximum likelihood (FIML).^15–17^ The proportion of missing data for each variable is described in the online supplemental tables S2 and S3 along with analyses comparing GHQ-28 at age 68–69 between those with and without missing data by religious attendance at ages 43, 60–64 and 68–69.

## Results

### Participants

Study members characteristics are presented in table 1. Approximately half of the participants were women, and one-third had no educational qualifications. The majority reported never attending religious services at all ages (82% at age 43, 81% at age 60–64 and 68% at age 68–69). There was a slight increase in the proportion of participants attending weekly and monthly at age 68–69 compared with ages 43 and 60–64.

**Table 1:** Participant characteristics (n=2125)

	N (%)/mean (SD)
Sex	
Male	1084 (51.0)
Female	1041 (49.0)
Education	
No qualifications	622 (30.9)
O-level	572 (28.4)
A-level	589 (29.3)
Higher education	230 (11.4)
GHQ-28	
Age 53	17.2 (9.4)
Age 60–64	16.4 (8.1)
Age 68–69	15.2 (7.9)
Religious attendance at age 43	
Never	1649 (82.2)
Less than monthly	49 (2.4)
Monthly	70 (3.5)
Weekly	237 (11.8)
Religious attendance at age 60–64	
Never	1496 (80.7)
Less than monthly	70 (3.8)
Monthly	72 (3.9)
Weekly	215 (11.6)
Religious attendance at age 68–69	
Never	1305 (68.0)
Less than monthly	215 (11.2)
Monthly	105 (5.5)
Weekly	294 (15.3)

GHQ-28, 28-item General Health Questionnaire.

### Associations between religious attendance and mental health

Table 2 shows the auto correlations over time for GHQ and for religious attendance; both track strongly over time (p≤0.001).

**Table 2:** Auto-correlation of measures of mental health and religious attendance across mid-life (n=2125)

General Health Questionnaire	Age 53	Age 60–64	Age 68–69
Age 53	1 (n=2902)		
Age 60–64	0.46 (n=2039)	1 (n=2190)	
Age 68–69	0.40 (n=1970)	0.54 (n=1829)	1 (n=2125)
**Religious attendance**	**Age 43**	**Age 60–64**	**Age 68–69**
Age 43	1 (n=3246)		
Age 60–64	0.62 (n=2281)	1 (n=2446)	
Age 68–69	0.59 (n=2178)	0.74 (n=2049)	1 (n=2395)

Values represent Pearson’s correlation coefficient, r: p<0.0001 for all correlations.

**Figure 3:**
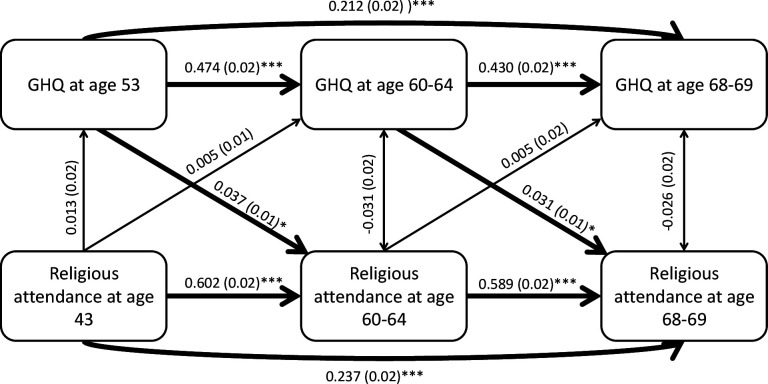
Auto-regressive cross-lagged model testing bi-directional associations between religious attendance and GHQ score, adjusted for gender and education. Figures represent standardised regression coefficients and SEs. GHQ, General Health Questionnaire. *p<0.05; **p<0.01: ***p<0.001.

Figure 3 presents the results of the auto-regressive cross-lagged analysis between religious attendance and mental health at three time points. Double-headed arrows represent cross-sectional associations between variables measured at the same time. As shown by the auto-correlations in table 2, previous religious attendance was strongly related to later attendance. Similarly, mental health at baseline was strongly associated with later mental health scores. Poorer mental health at age 53 and 60–64 was associated with more frequent religious attendance at age 60–64 (b=0.04; 95% CI: 0.02 to 0.06; p<0.05) and at age 68–69 (b=0.03; 95% CI: 0.02 to 0.06; p<0.05), respectively. There was no association at the 5% level between religious attendance and later or concurrent mental health. Sensitivity analysis which modelled religious attendance as a categorical variable, found the same pattern of associations (online supplemental table S4).

## Discussion

### Summary

This study tested bi-directional associations between religiosity and mental health using an autoregressive cross-lagged model to analyse data across 26 years. We found that religious attendance and mental health both track in middle-life and that poorer mental health is associated with more frequent religious attendance, but that religious attendance was not associated with subsequent mental health.

### Comparison with existing literature

Our findings do not support our stated hypotheses and are in contrast with the majority of previous research on religion and mental health.^5–7^ The most relevant study is by Li *et al*, who used longitudinal data from more than 48 000 female nurses in the USA to investigate associations between religious attendance and depressive symptoms, and change in religious service attendance and subsequent depression.^18^ They found that after 8 years, those with depression at baseline were less likely to attend religious services and that attending religious services was associated with a lower risk of depression. Our findings are also in contrast to those from a UK based longitudinal study where Aksoy *et al* found a positive association between religious attendance and mental health in a sample of over 50 000 people.^8^ Our novel findings are likely to be partially due to methodological differences. We employed a cross-lagged method to simultaneously assess reciprocal associations, whereas other studies examined bi-directional associations independently. It is also possible that differences in sample characteristics, such as age, ethnicity and religious affiliation, may explain the contrasting results. There is likely to be variation between the UK and USA, where most previous research on this topic has taken place, due to cultural differences in the relationship people have with religious organisations and in denomination between the two countries.^19^ For example, in this study 41% of study members reported their religious denomination as Protestant at age 36 (1982) compared with 58% of Americans in 1988. (See online supplemental table S5); previous comparisons of religious practices and beliefs between the UK and USA show that the USA is more religious on every measure.^20 21^

Furthermore, many studies on religion and mental health are with populations that are not generalisable to an adult community sample, for example, populations from clinical settings (such as those with a terminal illness) or children.^22–24^ Differences could also be due to the extended follow-up with birth cohort data; previous studies investigating bi-directional associations had follow-up times ranging from 10 weeks to 8 years.^7^ When comparing our findings, it is also important to consider the potential impact of cohort effects as previous research using data from repeated cross-sectional surveys and other cohort studies suggest a decline in the rates of religious attendance and belief in God across cohorts.^25 26^

A possible explanation for the findings is that participants may be using religious attendance as a coping mechanism in response to psychological distress. Religion is frequently reported as a coping mechanism for stressful life events, particularly in response to health problems.^27 28^ This process has been conceptualised as ‘religious coping’, which proposes that religious involvement can be protective of mental health by providing meaning in life and a framework for which to understand and deal with difficult situations, offering a sense of control and through the provision of social support and social cohesiveness through ritual and traditions.^29 30^ This hypothesis is supported by post-hoc analysis, which found that study members who were experiencing high levels of psychological distress at age 53 (a score of 24 or more on the GHQ-28 scale^31^ were more likely to report an increase in their religious attendance from age 43 to 60–64 compared with those with lower levels of distress (24.8% vs 51.9%). The same pattern was found when examining psychological distress at age 60–64 and an increase in attendance from this age to 68–69 (25.7% vs 38.5%). Details of this post-hoc analysis can be found in online supplemental table S6.

It is possible that being part of a religious community and regularly attending religious services confers access to emotional support, advice and practical help which are perceived to be beneficial for mental health.^32^ For example, Ross and Mirowsky found that people belonging to religious groups were more likely to talk about their problems than those in a non-religious groups.^33^ Some studies have found that religious beliefs and attendance are associated with reduced risk of loneliness via higher levels of social integration (measured by the size of their social networks and how often they see their friends and family) and social support.^34 35^

Despite there being several plausible mechanisms for how religious attendance may be beneficial for or protective or mental health, no such associations were found in this study. It is possible that the potential therapeutic effects of religious attendance may dissipate over time and therefore may not be evident several years later. Future research investigating study members who consistently attend religious attendance may help to understand this further.

### Strengths and limitations

The potential for reverse directionality has previously been identified in research relating to religiosity and health but has not been extensively researched.^7^ The results presented in this paper are the first examination of the bi-directional prospective associations between religious attendance and mental health in the UK. This analysis was enabled using an autoregressive cross-lagged model, which has many strengths but also several limitations.^36^ The main strength is that it can use repeated measures from cohort studies in order to better investigate reciprocal associations between two variables, in this case religious attendance and mental health, and obtain standardised estimates allowing a comparison of the associations in both directions.^14 37^ A limitation of the model is that the lagged effects reported in this study are a combination of between-person and within-person change with an additional cross-lagged influence of the factors investigated within the same individuals over time. However, despite the longitudinal nature of these analyses, it is still challenging to demonstrate a causal relationship between mental health and religious attendance. Furthermore, the time period was not equal between waves, and the measures of religious attendance and mental health were not always collected at the same time. This may lead to biases as cross-lagged models assume synchronicity between measures at the same time point and equidistance of time intervals.^38^ The phrasing of the questions to measure religious attendance varied slightly from wave to wave, and therefore it is possible this was interpreted differently by study members and may not measure the same construct.

Missing data are a common problem in longitudinal studies and can potentially lead to biased estimates and reduced statistical power. In this investigation, it was found that study members with missing data on religious attendance were more likely to have worse mental health compared with those with complete data (online supplemental table S3). Since it was possible that implementing complete case analysis would over-estimate associations between religiosity and mental health and well-being, missing data for exposure variables and covariates were handled by using FIML, allowing parameter estimation using all available data.^15–17^

This study only presents findings for religious attendance as these were the only religion-related variables for which comparable repeated measures were available. Future research should explore how different aspects of religion, such as beliefs and prayer, are associated with mental health. Although participants from NSHD provide a nationally representative sample, it is not possible to generalise these findings to younger generations, religions other than Christianity, or different cultures.^39^ The analyses presented in this paper should be extended accordingly.

### Implications and further research

Religious institutions can offer social and psychological support in times of distress by providing hope, meaning and social contact and support.^30^ Future research should explore this more explicitly by investigating whether religious attendance or other aspects of religiosity can moderate the impact of stressful life events on mental health. While it is not reasonable to advocate joining a religious group or taking up religious practices for those who do not identify as religious, further research using more in-depth measures into the mechanisms of how religion is associated with mental health could identify areas for intervention development. For example, research on aspects of religiosity such as gratitude, forgiveness and compassion has suggested some beneficial associations with mental health and are considered to be universal aspects of eudaemonic well-being that extend beyond religiosity.^40^

## Conclusion

Poor mental health was associated with later religious attendance but not vice versa. Future research should aim to confirm these novel findings and examine the processes through which mental health and religious attendance are associated.

**What is already known on this subject**

- There is some evidence that religious attendance is associated with benefits for mental health, with some studies suggesting that the relationship may be bi-directional, and that religious attendance is a marker for good health. Most of the previous research has taken place inthe USA and are not generalisable to the UK where the topic has remained unexplored.

**What this study adds**

- This study represents the first exploration of bi-directional associations between religious attendance and mental health using data across 26 years from a British birth cohort study. We found that religious attendance was not associated with later mental health, but that higher levels of depression and anxiety was associated with a later increase in religious attendance. As these findings are novel and contrary to most of the existing research, future work should aim to investigate potential mechanisms between mental health and religious attendance such as effect moderation of stressful life events.

## Data Availability

Data may be obtained from a third party and are not publicly available. Data sharing is dependent on the approval by the NSHD Data Sharing Committee, a data-sharing agreement being in place between UCL and the academic institution that employs the researcher, and LHA resources being available to meet the requests for data sharing. Metadata can be browsed on Skylark: https://skylark/ucl.ac.uk/Skylark.
