# Supplementary material for: Bi-directional associations between religious attendance and mental health: findings from a British birth cohort study
Source: J Epidemiol Community Health. 2021 Aug 5;76(2):190–5. doi: 10.1136/jech-2021-216943 (PMC8762020; doi:10.1136/jech-2021-216943)
Supplement: Supplementary data [file jech-2021-216943supp001.pdf]

Supplementary Tables

Table S1. Associations between mental health at age 68-69, and gender, education and social class

	**GHQ-28 (n=2125)**<br>b (95% CI)
Gender	
Male	Ref
Female	**0.11 (0.08,0.15)**
Education	
No qualification	Ref
O-levels	-0.02 (-0.07,0.03)
A-levels	**-0.07 (-0.13,-0.02)**
Higher education	**-0.09 (-0.17,-0.02)**
Social class (head of household) at age 53[1]	
Unskilled	Ref
Partly skilled	-0.01 (-0.12,0.14)
Skilled (manual)	-0.03 (-0.09,0.14)
Skilled (non-manual)	-0.01 (-0.11,0.14)
Intermediate	-0.01 (-0.12,0.11)
Professional	-0.06 (-0.20,0.07)

Mutually adjusted linear regression models
b = Unstandardized coefficients; CI= Confidence Intervals
[1] Head of household social class at 53 was derived from data available at age 53 (or using ages 43, 36 or 26 if missing). This used the study member's social class if they were male and partner's social class if female, categorised into professional, intermediate, skilled (non-manual), skilled (manual), semi-skilled manual or unskilled, according to the UK Registrar General's Classification of Occupations (Office of Population. Classification of Occupations HMSO. In Censuses and Surveys, ed. London, 1970.)

Table S2. The proportion of complete data for each variable (n=2125)

Variable	**N (%)**
**GHQ-28**	
Age 60-64	1829 (86.1)
Age 53	1970 (92.7)
Age 68-69	2125 (100.0)
**Religious practice**	
Age 43	2005 (94.4)
Age 60-64	1853 (87.2)
Age 68-69	1919 (90.3)
**Socio-economic variables**	
Gender	2125 (100.0)
Education by age 26	2013 (94.7)
Social class at age 53 (imputed from age 43, 36 or 26 if missing)	2114 (99.5)

GHQ-28: 28-item General Health Questionnaire

Table S3. Association between missing data and GHQ-28 at age 68-69

Variable	Observation	Mean (SD)	P (t-test)
**Religious practice at age 43**			
Complete data	2005	15.18	0.9
Missing data	120	15.07	
**Religious practice at age 60-64**			
Complete data	1853	14.84	<0.001
Missing data	272	17.46	
**Religious practice at age 68-69**			
Complete data	1919	14.92	<0.001
Missing data	206	17.56	

GHQ-28: 28-item General Health Questionnaire; SD: Standard Deviation

Table S4. Sensitivity analysis of auto-regressive cross-lagged model of religious attendance (categorical) and mental health from age 43 to 68-69

	Religious attendance modelled as a continuous variable	Religious attendance modelled as a categorical variable
	**β (95% CI)**	**B (95% CI)**
GHQ-28 at age 68-69		
ON GHQ-28 at age 60-64	**0.430 (0.395,0.464)**	**0.227 (0.227,0.237)**
ON Attendance at age 60-64	0.005 (-0.018,0.028)	0.002 (-0.045,0.049)
WITH Attendance at age 68-69	-0.026 (-0.066,0.013)	0.007 (-0.086,-0.079)
ON GHQ-28 at age 53	**0.212 (0.177,0.247)**	**0.399 (0.378,0.420)**
Attendance at age 68-69		
ON GHQ-28 at age 60-64	**0.031 (0.013,0.049)**	**0.021 (0.004,0.039)**
ON Attendance at age 60-64	**0.589 (0.561,0.618)**	**0.791 (0.715,0.868)**
ON Attendance at age 43	**0.237 (0.205,0.268)**	**0.142 (0.058,0.226)**
GHQ-28 at age 60-64		
ON GHQ-28 at age 53	**0.474 (0.444,0.504)**	**0.042 (0.027,0.057)**
ON Attendance at age 43	0.005 (-0.017,0.027)	0.000 (-0.011,0.012)
WITH Attendance at age 60-64	-0.031 (-0.071,0.009)	0.007 (-0.030,0.045)
Attendance at age 60-64		
ON Attendance at age 43	**0.602 (0.577,0.626)**	**0.803 (0.769,0.837)**
ON GHQ-28 at age 53	**0.037 (0.015,0.058)**	**0.054 (0.008,0.100)**
**GHQ-28 at age 53**		
ON Attendance at age 43	0.013 (-0.024,0.050)	0.027 (-0.030,0.084)

β = Standardized beta coefficients; CI= Confidence Intervals

Table S5. Religious denomination of study members at age 36.

	**n (%)**
No religious belief	1250 (38.4)
Protestant	1342 (41.2)
Roman Catholic	213 (6.5)
Other	454 (13.9)

Table S6. Association between psychological distress and subsequent increase in the frequency of religious attendance.

	**The difference in religious attendance from age 43 to age 60-64 (n=3142)**				
	**Decrease n (%)**	**No change n (%)**	**Increase n (%)**	**P (χ^2)**	**P (trend)**
**Psychological distress (age 53)**					
Low	151 (7.0)	1417 (68.2)	535 (24.8)	**<0.001**	**<0.001**
High	40 (4.1)	434 (44.1)	511 (51.9)		
	**The difference in religious attendance from age 60-64 to age 68-69 (n=2393)**				
	**Decrease n (%)**	**No change n (%)**	**Increase n (%)**		
**Psychological distress (age 60-64)**					
Low	52 (3.1)	1181 (71.4)	427 (25.7)	**<0.001**	**<0.001**
High	14 (1.9)	437 (59.6)	282 (38.8)		

Psychological distress was assessed using the GHQ-28. Scores of 24 and more were considered to indicate high levels of psychological distress.

Kaushal A, *et al. J Epidemiol Community Health* 2021; 76:190–195. doi: 10.1136/jech-2021-216943
